# IMPLEMENTING REACH COMMUNITY IN AN INTEGRATED HEALTH SYSTEM: IMPACT AND LESSONS LEARNED

**DOI:** 10.1093/geroni/igae098.1689

**Published:** 2024-12-31

**Authors:** Tamara Herrick

**Affiliations:** MaineHealth, Portland, Maine, United States

## Abstract

This project examined the implementation of the Resources for Enhancing Alzheimer’s Caregiver Health (REACH) Community program within primary care and geriatrics settings, facilitated by Licensed Clinical Social Workers (LCSWs). The integration of LCSWs into the healthcare system offers a unique opportunity to provide comprehensive care by addressing both medical and psychosocial aspects of dementia caregiving. Their expertise in psychosocial assessment and intervention enables holistic care that addresses the multifaceted needs of care partners and their families. REACH Community is an evidence-based intervention for care partners of individuals living with dementia that focuses on safety, care partner health and emotional well-being, and behavior management. The intervention consists of four core sessions one-on-one with care partners, with additional sessions as an option if needed. Sessions can be delivered face-to-face, by telephone, or telehealth. With the emergence of COVID-19, all sessions transitioned to remote delivery via telehealth Zoom platform or telephone. Pretest posttest design assessed program impact. Key outcome measures included: Caregiver Frustration, caregiver well-being (Zarit Burden Inventory), and mood (PH-Q4). Pre-posttest results indicated that participants had an improvement in well-being, mood, and caregiver frustration following participation in the REACH Community program. The evaluation contributed valuable insights into the feasibility and effectiveness of integrating the REACH Community program into primary care and geriatrics settings utilizing LCSWs. We continue to offer the program in primary care and geriatrics center settings and REACH Community is part of our growing toolbox of evidence-based programs providing support to care partners of people living with dementia.

